# Extreme fertilization bias towards freshly inseminated sperm in a species exhibiting prolonged female sperm storage

**DOI:** 10.1098/rsos.172195

**Published:** 2018-03-07

**Authors:** Clelia Gasparini, Emma Daymond, Jonathan P. Evans

**Affiliations:** Centre for Evolutionary Biology, School of Biological Sciences, University of Western Australia, Crawley 6009, Australia

**Keywords:** sperm precedence, sperm ageing, sexual selection, female sperm storage

## Abstract

The storage of sperm by females across successive reproductive cycles is well documented in internal fertilizers, yet the fate of stored sperm when they compete with ‘new’ sperm to fertilize a female's eggs has rarely been considered. This gap in our understanding is likely due to the logistical difficulties of controlling behavioural interactions during or after mating, which in turn may influence how many sperm are inseminated and how stored sperm are ultimately used during successive bouts of sperm competition with freshly inseminated sperm. Here, we use artificial insemination (AI) in guppies (*Poecilia reticulata*), a polyandrous live-bearing poeciliid fish exhibiting prolonged sperm storage by females, to overcome these challenges. The use of AI enables us to control potential differential maternal effects (e.g. behaviourally mediated cryptic female choice) and specifically test for post-copulatory paternity biases that favour either stored or fresh sperm when they compete to fertilize eggs. Our paternity analyses revealed the almost complete dominance of freshly inseminated sperm over stored sperm, supporting previous studies reporting similar patterns following natural matings across successive brood cycles. However, our use of AI, which excluded behavioural interactions between males and females, most likely generated a far stronger pattern of fresh sperm precedence compared with those reported in previous studies, possibly implicating ‘cryptic' forms of selection by females that may sometimes bolster the success of stored sperm.

## 1. Introduction

The capacity for females to store sperm after mating is widespread in internally fertilizing species [1]. Female sperm storage (FSS), where viable sperm are maintained within the female's reproductive tract, is an important physiological adaptation in many species with important evolutionary consequences for animal mating systems [1–3]. In particular, FSS means that mating can be temporally separated from fertilization, thereby allowing females to control the timing of offspring production and increasing the opportunity for post-copulatory sexual selection (i.e. sperm competition, where ejaculates from two or more males compete to fertilize eggs [4], and cryptic female choice (CFC), where females influence the outcome of these contests [5]).

**Figure 1.**
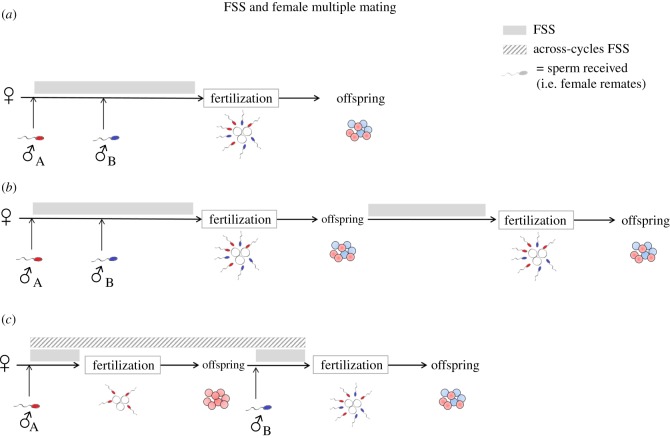
Female sperm storage (FSS) and female multiple matings: a schematic of hypothetical scenarios when both FSS and multiple mating occurs: (*a*) there is ‘FSS' but does not encompass different reproductive cycles (i.e. sperm are not further stored after fertilization); (*b*) there is ‘prolonged FSS’ with multiple broods produced from the initial matings, without remating by the female; (*c*) there is ‘across-cycles FSS' and sperm received in different reproductive cycles compete to fertilize eggs.

Although FSS typically involves sperm being stored for a variable period of time (hours, days and even years) until they are used for fertilization, in some species sperm can also be stored after fertilization, and thus FSS can span several successive reproductive cycles (hereafter ‘across-cycles FSS'). This allows females to re-mate and obtain fresh sperm when stored sperm are still present in the female storage structure (see figure 1 for a visualization of FSS within and across cycles). In reptiles [6,7] and fishes [8,9], for example, females often produce offspring using both fresh sperm and those stored from previous reproductive cycles. Although sperm storage can be costly [10,11], it is beneficial for females under many circumstances. For example, FSS allows females to produce numerous broods in the absence of males and is therefore an important trait for invasion success of new habitats [12,13], and to maintain within-brood genetic variability when mate availability is low. In addition, across-cycles FSS may have important implications for sperm competition, potentially influencing fertilization dynamics when fresh sperm compete with previously stored sperm (figure 1). For the most part, however, patterns of sperm precedence (i.e. biases in competitive fertilization success that favour either the first or last male to mate with a female) are reported only when two or more males mate successively with a female within a single reproductive cycle (e.g. [14–17]). By contrast, surprisingly few studies have investigated how stored sperm (from previous reproductive cycles) compete with freshly inseminated sperm, which is highly relevant in species exhibiting across-cycles FSS. The limited evidence published to date suggests that sperm inseminated in the most recent reproductive cycle gain disproportionately high fertilization success when competing with previously stored sperm [6,8,9,18].

The guppy (*Poecilia reticulata*) provides an ideal species for studying competitive fertilization success when fresh sperm compete with previously stored sperm. Guppies are live-bearing freshwater fish with internal fertilization in which females are able to store sperm arising from a single copulation for up to eight successive reproductive cycles (each cycle approx. one month) [9,19,20]. Female guppies are highly polyandrous [21] and sperm competition plays a central role in their mating system [22]. Moreover, the presence of across-cycles FSS, coupled with high rates of multiple mating (including those attributable to ‘forced' unsolicited matings [23]), means that sperm competition will frequently involve ejaculates inseminated during different reproductive cycles. Although sperm competition has been studied extensively in guppies [22], only a handful of studies have considered the implications of FSS for competitive fertilization success. Recently, Devigili *et al.* [24] studied how FSS affects sperm competition success across different broods when sperm are fresh or stored, but to our knowledge there are only two studies in the 1950s documenting patterns of sperm precedence for ejaculates inseminated in successive reproductive cycles (i.e. one fresh, one stored). These two pioneering studies documented a strong bias towards freshly inseminated sperm, although both have limitations in terms of small sample size (fewer than 10 fish) and the systematic use of mutant male strains as stored sperm donors and wild-type males as fresh sperm donors [8,18].

Here we investigate patterns of sperm precedence in guppies when fresh sperm compete with stored sperm from the preceding reproductive cycle. Specifically, we use paternity analyses to estimate the fertilization success of sperm inseminated into virgin females during an initial reproductive cycle when they compete with rival male sperm inseminated in the successive cycle, *ca* one month later. Importantly, we take the precaution of using artificial insemination (AI) in order to control (i) the timing of matings and (ii) the relative number of sperm inseminated during successive brood cycles (both within and among replicates). The use of AI also allows us to exclude any effects attributable to behavioural interactions between males and females (e.g. differential sperm utilization based on the female's perception of attractiveness [25]). In this way, we are able to focus exclusively on how sperm inseminated during successive reproductive cycles compete with each other. To minimize stochastic effects associated with the assignment of competitor males [26], we created a fully reciprocal design in which sperm from two different males were used under reciprocal experimental conditions (as ‘stored' or ‘fresh'). We then estimated competitive fertilization success by calculating the relative paternity success of males assigned to the fresh and stored sperm treatments.

## 2. Material and methods

### 2.1. Animals

The guppies used in this experiment were laboratory-reared descendants of wild-caught fish from Alligator Creek River, Queensland, Australia. Fish were maintained in tanks with equal sex ratio on a 12L : 12D cycle at 26(±1)°C, and fed with a mix of *Artemia nauplii* and commercial dry food. Males used in the experiment were selected randomly from stock populations of known age (six–eight months old), while virgin females were reared from birth in single-sex tanks and used when six months old.

### 2.2. Experimental overview

**Figure 2.**
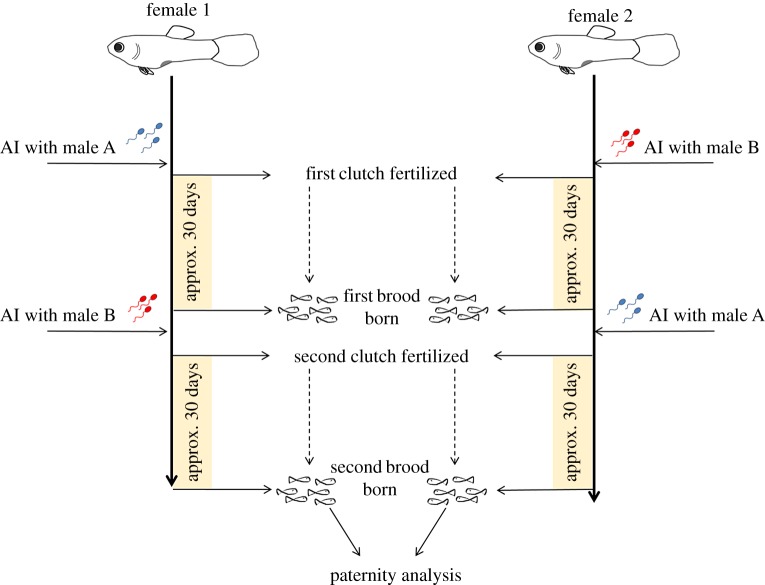
A schematic of the experimental design. Only one block (out of 17) is depicted for simplicity. Each block consists of two virgin females (female 1 and female 2) and two males (male A and male B). Paternity analyses were conducted on the second brood (as the first brood was sired exclusively by the first male). AI, artificial insemination.

Each experimental block consisted of a fully reciprocal design in which two males (labelled A and B in figure 2) and two virgin females (labelled 1 and 2 in figure 2) were used. Initially, each female was inseminated (see Artificial inseminations section) with the sperm (20 bundles, see below) from one of the two males (female 1 with male A and female 2 with male B). One to three days after the birth of the first brood (approx. 30 days after the initial insemination) each female was artificially inseminated with the same quantity of sperm (20 bundles) from the other male in the block (female 1 with male B and female 2 with male A). This reciprocal design ensured that the sperm from the same male were used in both conditions (i.e. stored in one female and fresh in the other female). We conducted 17 of these blocks.

### 2.3. Artificial inseminations

Males and females were individually isolated in small 4 l plastic tanks for a week prior to the experiment. Sperm were collected following established protocols [27]. Twenty sperm bundles (each containing approx. 21 000 sperm [27]) from the assigned male were used to inseminate each female. Immediately after insemination, the female was returned to her original tank where she was left in isolation until she produced her first brood. The female was then inseminated with the other male's sperm between 1 and 3 days after producing offspring. We chose this period for the second inseminations because it coincides with the period of maximum female sexual receptivity, where natural matings peak between 1 and 3 days after parturition [23,28]. This interval also coincides with the period when sperm from previous reproductive cycles are released from the female's sperm storage sites [29]; mixed-paternity broods arising from fertilization by stored and freshly inseminated sperm have been reported as a result of natural matings occurring between 1 and 6 days after the birth of the first brood [8]. Therefore, the timeframe we selected (1–3 days, mean: 2.08 ± 0.76 s.d.) ensured that both stored and fresh sperm would have had the potential to fertilize the female's eggs.

### 2.4. Paternity analysis

Fin clips from males and females and the whole body of the newborns were used for DNA extraction. DNA was extracted using EDNA HISPEX™ Tissue kit (Fisher Biotec) and amplified using standard polymerase chain reaction protocols (for details, see [27]). Products were processed on an ABI3730 Sequencer and analysed using GENEMARKER software (Soft Genetics). Paternity was assigned using five microsatellites (TTA, AGAT11, Kond15, Kond21, Pret46, GenBank accession numbers: AF164205, BV097141, AF368429, AF368430, AF127242 respectively). The software CERVUS (v. 3.0.7, available at http://www.fieldgenetics.com) was used to identify the most likely father with 80% confidence.

### 2.5. Statistical analyses

Paternity success was analysed using a generalized linear mixed-effects model with a binomial error distribution and a logit-link function (using ‘lme4' package). Only broods comprising three or more babies were considered in these analyses. The model included treatment (fresh or stored) as a fixed factor and block ID as a random factor. The model was also weighted by the total number of offspring (whose paternity was assigned) in each brood. The *p*-value for the fixed effect was obtained using the ‘ANOVA' function in the ‘car' package. All statistical analyses were performed in R v. 3.3 [30].

## 3. Results

Three out of the *n *= 17 blocks were excluded from the analysis due to the high similarity between the two potential sires which made paternity assignment unreliable. Therefore, a total of 14 blocks were used in the final analysis, comprising a total of 28 males, 28 females and 224 offspring. Females produced a mean (±s.d.) of 5.93 ± 2.79 babies (range 1–11) in their first brood, and 7.96 ± 2.76 babies (range 3–15) in their second broods. This difference between a female's first and second broods was statistically significant (paired *t*-test, *t*_27_ = 3.988, *p* < 0.001). Paternity was successfully assigned to 191 out of 224 newborns (86%) for a total of 27 families. In 25 of these families, the fresh sperm sired the entire brood, while in two families offspring arose from fertilizations by both fresh and stored sperm (stored/fresh: 2/7 and 1/5, respectively). Overall, the mean proportion of offspring arising from fertilizations by fresh sperm was 0.986 (± 0.052 s.d.), which clearly represents a highly significant fertilization advantage towards fresh sperm (*χ*^2^ = 15.154, *p* < 0.001).

## 4. Discussion

Our study of sperm precedence in a species exhibiting among-cycles FSS reveals an extreme paternity bias that favours freshly inseminated sperm over those stored from a previous reproductive cycle. To the best of our knowledge, this is the first study to have addressed this question using an experimental design that precludes potentially confounding factors arising from behavioural interactions between the sexes (e.g. females ‘trading up’ or differentially bolstering the insemination success of ‘preferred’ males [25,31]). Our findings are in general agreement with previous studies reporting that stored sperm are less competitive than freshly inseminated sperm [6,18]. However, the extent of the paternity bias in our experiment was surprising; we found almost complete sperm precedence towards freshly inseminated sperm (approx. 99% after AI), which contrasts with previous work (based on natural matings) revealing precedence towards fresh sperm at about 83% and 60% when natural matings occurred 1 and 4 days after birth of the first brood, respectively [8]. Below, we consider a number of (non-mutually exclusive) factors that may account for the extreme paternity bias observed in this study.

One explanation for the low fertilization success of stored sperm is that sperm numbers may have been depleted to ineffective levels during sperm storage. It is possible that AI may have exacerbated such a decline in sperm number, thus accounting for the extreme bias in competitive fertilization success towards freshly inseminated sperm compared to previous studies using natural mating (see above). However, based on our knowledge of the guppy's reproductive biology, and prior work on the focal population, this explanation is somewhat simplistic. For example, we know that in the focal population used for the current study, AIs involving the same number of sperm that were used here have resulted in females producing up to five consecutive broods, with 31 out of 36 females successfully producing a second brood without any further sperm supplementation (brood sizes of the two successive broods not significantly different, paired *t*-test *t*_30_ = 0.594, *p* = 0.557, C. Gasparini 2017, unpublished data). A similar study employing AI on a different guppy population revealed no significant effect of the number of sperm inseminated on the probability of producing a brood, even when sperm numbers were reduced tenfold compared to the numbers used in the present study [32]. However, it is important to note that these prior AI studies did not involve sperm competition. We know that the relative number of sperm from rival males is an important determinant of sperm competition success in guppies [33], and thus any decline in sperm number during sperm storage may explain the extreme fresh sperm precedence towards fresh sperm observed in our study (see also [19]).

A second explanation for our findings includes the possibility that stored sperm undergo a reduction in sperm quality during storage. We know that sperm can be prone to a range of deleterious effects associated with ageing, exhibiting reductions in performance, impaired DNA integrity and various degrees of cellular damage as they age inside the female's sperm storage organs [34,35]. Such detrimental effects of sperm ageing during storage may further exacerbate any reductions in sperm number, resulting in sperm with low competitive ability. It is also possible that any impairment in sperm quality attributable to sperm ageing may influence embryo mortality [34,36]. Guppies are livebearers and females carry developing embryos for approximately one month before they are born. If embryos arising from fertilizations by stored sperm exhibited higher mortality (e.g. through embryo death or reabsorption), we might expect to see an underestimation of fertilization success of stored sperm (which would be reflected by the reduction in paternity success observed in our study). However, in our study, the second brood was larger than the first one, so we can confidently exclude differential mortality of embryos as a factor explaining our observed patterns of paternity. The reason behind this difference in brood size between the first and the second brood is unclear. We suspect that it is unlikely to be attributable to changes in female size, as female growth from one cycle to the next is minimal and, therefore, unlikely to account for the difference in offspring number between successive broods [37]. We also suspect that this difference is unlikely to be due to multiple paternity in the second brood, as this was extremely low in our experiment [38]. One possible explanation for the difference in brood size is that non-sperm components (i.e. seminal fluid) in the initial insemination may have stimulated egg production in the successive cycle, leading to a larger number of eggs available for fertilization during the second insemination.

A further possible explanation for our findings is that females may exert post-copulatory fertilization biases (i.e. CFC) that favour fresh sperm over stored sperm when they compete to fertilize eggs [5,39]. If the offspring arising from stored sperm are of lower quality [40], for example, due to the deleterious effects of ageing (see above), females would benefit by exerting CFC for fresh sperm. In guppies, we do not know whether offspring arising from fertilizations by stored sperm are of lower quality than those arising from fresh sperm, although the observation that a single pregnant female can establish a viable population [12,13] suggests that offspring quality does not decline appreciably across cycles of FSS. Similarly, in natural populations stored sperm have been shown to be fertile well beyond the male's lifetime, suggesting that strong CFC against stored sperm is unlikely [9]. It seems improbable, therefore, that CFC favouring fresh sperm could explain the extreme paternity bias towards fresh sperm seen in our study. We, therefore, find that CFC for fresh sperm alone is unlikely to explain the pattern we found, particularly in the light of previous work showing that stored sperm arising from natural matings continue to fertilize eggs over different reproductive cycles even in presence of freshly inseminated sperm [8,18,20].

One important difference between the present experiment and previous studies exploring this topic is that we used AI rather than natural matings. We did this to ensure that we controlled for any behavioural interactions that may have biased patterns of sperm precedence independently of the effects of sperm storage. For example, female guppies have been shown to be more willing to re-mate when new males are superior (e.g. more attractive) than their previous mate [31], and females will differentially accept more sperm from preferred males [25,41]. Thus, behavioural interactions have the potential to confound patterns of paternity when fresh sperm compete with previously stored sperm. However, it could be argued that behavioural interactions between males and females are required to activate stored sperm. However, this argument does not apply to guppies, as females kept in isolation from males are able to produce several successive broods [12,19,20,24], indicating that fertilization by stored sperm occurs in the absence of any behavioural or visual interaction with males. Furthermore, in experimental designs using natural matings, males and females often mate more than once (consensually or through forced copulations). Under these conditions, females are likely to store considerably more sperm from the first mating (in the first reproductive cycle). In future work, it would be interesting to determine whether experimental changes in ejaculate size during the initial AIs (first reproductive cycle) reduce the fresh sperm advantage during subsequent cycles.

In conclusion, our experiment is one of only a handful of studies that investigate across-cycles FSS. We report an extreme bias in paternity towards males contributing fresh sperm, which is likely due in part to the use of AIs, which excluded behavioural interactions between males and females. Consequently, we suggest that behavioural interactions may play an important role in the evolution and maintenance of FSS. Future studies would benefit by establishing the relative importance of such interactions, along with the effects of sperm number and sperm quality in generating the patterns of fresh sperm precedence reported here and elsewhere.

## Data accessibility

The data associated to this study are available on the Dryad online data repository at: http://dx.doi.org/10.5061/dryad.7v1hq [42]

## References

[1] Orr TJ, Brennan PLR 2015 Sperm storage: distinguishing selective processes and evaluating criteria. Trends Ecol. Evol. 30, 261–272. (doi:10.1016/j.tree.2015.03.006) 25843274 10.1016/j.tree.2015.03.006

[2] Orr TJ, Zuk M 2012 Sperm storage. Curr. Biol. 22, R8–R10. (doi:10.1016/j.cub.2011.11.003) 22240479 10.1016/j.cub.2011.11.003

[3] Birkhead TR, Møller AP 1993 Sexual selection and the temporal separation of reproductive events: sperm storage data from reptiles, birds and mammals. Biol. J. Linnean Soc. 50, 295–311. (doi:10.1006/bijl.1993.1060)

[4] Parker GA 1970 Sperm competition and its evolutionary consequences in the insects. Biol. Rev. 45, 525–567. (doi:10.1111/j.1469-185X.1970.tb01176.x)

[5] Eberhard WG 1996 Female control: sexual selection by cryptic female choice. Princeton, NJ: Princeton University Press.

[6] Uller T, Schwartz T, Koglin T, Olsson M 2013 Sperm storage and sperm competition across ovarian cycles in the dragon lizard, *Ctenophorus fordi*. J. Exp. Zool. Part A 319, 404–408. (doi:10.1002/jez.1803) 10.1002/jez.1803 23744523

[7] Olsson M, Schwartz T, Uller T, Healey M 2007 Sons are made from old stores: sperm storage effects on sex ratio in a lizard. Biol. Lett. 3, 491–493. (doi:10.1098/rsbl.2007.0196) 17650477 10.1098/rsbl.2007.0196 PMC2391176

[8] Rosenthal HL 1952 Observations on reproduction of the poeciliid *Lebistes reticulatus* (Peters). Biol. Bull. 102, 30–38. (doi:10.2307/1538621)

[9] López-Sepulcre A, Gordon SP, Paterson IG, Bentzen P, Reznick DN 2013 Beyond lifetime reproductive success: the posthumous reproductive dynamics of male Trinidadian guppies. Proc. R. Soc. B 280, 20131116 (doi:10.1098/rspb.2013.1116) 10.1098/rspb.2013.1116 PMC3774245 23740786

[10] Baer B, Armitage SAO, Boomsma JJ 2006 Sperm storage induces an immunity cost in ants. Nature 441, 872–875. (doi:10.1038/nature04698) 16778889 10.1038/nature04698

[11] McNamara KB, Van Lieshout E, Simmons LW 2014 Females suffer a reduction in the viability of stored sperm following an immune challenge. J. Evol. Biol. 27, 133–140. (doi:10.1111/jeb.12278) 24251540 10.1111/jeb.12278

[12] Deacon AE, Barbosa M, Magurran AE 2014 Forced monogamy in a multiply mating species does not impede colonisation success. BMC Ecol. 14, 18 (doi:10.1186/1472-6785-14-18) 24925225 10.1186/1472-6785-14-18 PMC4067062

[13] Deacon AE, Ramnarine IW, Magurran AE 2011 How reproductive ecology contributes to the spread of a globally invasive fish. PLoS ONE 6, e24416 (doi:10.1371/journal.pone.0024416) 21957449 10.1371/journal.pone.0024416 PMC3176282

[14] Xu J, Wang Q 2010 Mechanisms of last male precedence in a moth: sperm displacement at ejaculation and storage sites. Behav. Ecol. 21, 714–721. (doi:10.1093/beheco/arq044)

[15] Evans JP, Magurran AE 2001 Patterns of sperm precedence and predictors of paternity in the Trinidadian guppy. Proc. R. Soc. Lond. B 268, 719–724. (doi:10.1098/rspb.2000.1577) 10.1098/rspb.2000.1577 PMC1088661 11321060

[16] Birkhead TR, Wishart GJ, Biggins JD 1995 Sperm precedence in the domestic-fowl. Proc. R. Soc. Lond. B 261, 285–292. (doi:10.1098/rspb.1995.0149)

[17] Squires ZE, Wong BBM, Norman MD, Stuart-Fox D 2015 Last male sperm precedence in a polygamous squid. Biol. J. Linnean Soc. 116, 277–287. (doi:10.1111/bij.12590)

[18] Hildemann WH, Wagner ED 1954 Intraspecific sperm competition in *Lebistes reticulatus*. Am. Nat. 88, 87–91. (doi:10.1086/281813)

[19] Winge Ø 1937 Succession of broods in *Lebistes*. Nature 140, 467 (doi:10.1038/140467b0)

[20] Constantz GD 1984 Sperm competition in poeciliid fishes. In Sperm competition and the evolution of animal mating systems (ed. Smith RL), pp. 465–485. Orlando, FL: Academic Press.

[21] Hain TJA, Neff BD 2007 Multiple paternity and kin recognition mechanisms in a guppy population. Mol. Ecol. 16, 3938–3946. (doi:10.1111/j.1365-294X.2007.03443.x) 17850555 10.1111/j.1365-294X.2007.03443.x

[22] Evans JP, Pilastro A 2011 Postcopulatory sexual selection. In Ecology and evolution of poeciliid fishes (eds Evans JP, Pilastro A, Schlupp I), pp. 197--208 Chicago, IL: University of Chicago Press.

[23] Houde AE 1997 Sex, color and mate choice in guppies. Princeton, NJ: Princeton University Press.

[24] Devigili A, Di Nisio A, Grapputo A, Pilastro A 2016 Directional postcopulatory sexual selection is associated with female sperm storage in Trinidadian guppies. Evolution 70, 1829–1843. (doi:10.1111/evo.12989) 27345870 10.1111/evo.12989

[25] Pilastro A, Simonato M, Bisazza A, Evans JP 2004 Cryptic female preference for colorful males in guppies. Evolution 58, 665–669. (doi:10.1111/j.0014-3820.2004.tb01690.x) 15119451

[26] García-González F 2008 The relative nature of fertilization success: implications for the study of post-copulatory sexual selection. BMC Evol. Biol. 8, 140 (doi:10.1186/1471-2148-8-140) 18474087 10.1186/1471-2148-8-140 PMC2408597

[27] Gasparini C, Dosselli R, Evans JP 2017 Sperm storage by males causes changes in sperm phenotype and influences the reproductive fitness of males and their sons. Evol. Lett. 1, 16–25. (doi:10.1002/evl3.2) 10.1002/evl3.2 PMC6121797 30283635

[28] Liley NR 1966 Ethological isolating mechanisms in four sympatric species of Poeciliid fishes. Behav. Suppl. 13, 1–197.

[29] Jalabert B, Billard R 1969 Étude ultrastructurale du sité de conversation des spermatozoides dans l'ovaire de *Poecilia reticulata* (Poisson, Téléostéen). Ann. Biol. Anim. Biochim. Biophys. 2, 273–280. (doi:10.1051/rnd:19690209)

[30] R Development Core Team. 2016 R: a language and environment for statistical computing.

[31] Pitcher TE, Neff BD, Rodd FH, Rowe L 2003 Multiple mating and sequential mate choice in guppies: females trade up. Proc. R. Soc. Lond. B 270, 1623–1629. (doi:10.1098/rspb.2002.2280) 10.1098/rspb.2002.2280 PMC1691420 12908984

[32] Pilastro A, Gasparini C, Boschetto C, Evans JP 2008 Colorful male guppies do not provide females with fecundity benefits. Behav. Ecol. 19, 374–381. (doi:10.1093/beheco/arm140)

[33] Boschetto C, Gasparini C, Pilastro A 2011 Sperm number and velocity affect sperm competition success in the guppy (*Poecilia reticulata*). Behav. Ecol. Sociobiol. 65, 813–821. (doi:10.1007/s00265-010-1085-y)

[34] Reinhardt K 2007 Evolutionary consequences of sperm cell aging. Q. Rev. Biol. 82, 375–393. (doi:10.1086/522811) 18217528 10.1086/522811

[35] Reinhardt K, Ribou A-C 2013 Females become infertile as the stored sperm's oxygen radicals increase. Sci. Rep. 3, 2888 (doi:10.1038/srep02888)

[36] Tarin JJ, Perez-Albala S, Cano A 2000 Consequences on offspring of abnormal function in ageing gametes. Hum. Reprod. Update 6, 532–549. (doi:10.1093/humupd/6.6.532) 11129687 10.1093/humupd/6.6.532

[37] Evans JP, Gasparini C 2013 The genetic basis of female multiple mating in a polyandrous livebearing fish. Ecol. Evol. 3, 61–66. (doi:10.1002/ece3.435) 10.1002/ece3.435 PMC3568843 23403856

[38] Evans JP, Magurran AE 2000 Multiple benefits of multiple mating in guppies. Proc. Natl Acad. Sci. USA 97, 10 074–10 076. (doi:10.1073/pnas.180207297) 10.1073/pnas.180207297 PMC27698 10954750

[39] Firman RC, Gasparini C, Manier MK, Pizzari T 2017 Postmating female control: 20 years of cryptic female choice. Trends Ecol. Evol. 32, 368–382. (doi:10.1016/j.tree.2017.02.010) 28318651 10.1016/j.tree.2017.02.010 PMC5511330

[40] Pizzari T, Dean R, Pacey A, Moore H, Bonsall MB 2008 The evolutionary ecology of pre- and post-meiotic sperm senescence. Trends Ecol. Evol. 23, 131–140. (doi:10.1016/j.tree.2007.12.003) 18280006 10.1016/j.tree.2007.12.003

[41] Pilastro A, Mandelli M, Gasparini C, Dadda M, Bisazza A 2007 Copulation duration, insemination efficiency and male attractiveness in guppies. Anim. Behav. 74, 321–328. (doi:10.1016/j.anbehav.2006.09.016)

[42] Gasparini C, Daymond E, Evans JP 2018 Data from: Extreme fertilization bias towards freshly inseminated sperm in a species exhibiting prolonged female sperm storage Dryad Digital Repository (doi:10.5061/dryad.7v1hq) 10.1098/rsos.172195 PMC5882737 29657813

